# An Observation Scheduling Approach Based on Task Clustering for High-Altitude Airship

**DOI:** 10.3390/s22052050

**Published:** 2022-03-06

**Authors:** Jiawei Chen, Qizhang Luo, Guohua Wu

**Affiliations:** 1 School of Computational Science and Engineering, Georgia Institute of Technology, Atlanta, GA 30332, USA; jchen897@gatech.edu; 2 School of Traffic & Transportation Engineering, Central South University, Changsha 410075, China; guohuawu@csu.edu.cn; 3 Department of Electrical & Computer Engineering, National University of Singapore, Singapore 119260, Singapore

**Keywords:** airship, observation scheduling, task clustering, heuristic algorithm, ant colony optimization

## Abstract

Airship-based Earth observation is of great significance in many fields such as disaster rescue and environment monitoring. To facilitate efficient observation of high-altitude airships (HAA), a high-quality observation scheduling approach is crucial. This paper considers the scheduling of the imaging sensor and proposes a hierarchical observation scheduling approach based on task clustering (SA-TC). The original observation scheduling problem of HAA is transformed into three sub-problems (i.e., task clustering, sensor scheduling, and cruise path planning) and these sub-problems are respectively solved by three stages of the proposed SA-TC. Specifically, a novel heuristic algorithm integrating an improved ant colony optimization and the backtracking strategy is proposed to address the task clustering problem. The 2-opt local search is embedded into a heuristic algorithm to solve the sensor scheduling problem and the improved ant colony optimization is also implemented to solve the cruise path planning problem. Finally, extensive simulation experiments are conducted to verify the superiority of the proposed approach. Besides, the performance of the three algorithms for solving the three sub-problems are further analyzed on instances with different scales.

## 1. Introduction

Earth observation techniques play a key role in environmental surveillance, intelligence reconnaissance, and disaster relief [1]. As a new kind of Earth observation platform, the high-altitude airship (HAA) has many advantages [2,3]. For instance, compared with the traditional unmanned aerial vehicle (UAV), HAA can fly continuously for several months, which makes HAA observe and acquire data uninterruptedly for a long period. In comparison with the Earth observation satellite (EOS) that only can observe ground targets within specified time windows [4,5], HAA has stronger abilities of rapid response and maneuverability. In addition, HAA has a lower cost in deployment, operation, and control. Therefore, HAA has become an ideal alternative Earth observation platform.

The practical applications of HAA in civilian fields have been investigated by several scientists and groups. For example, Belozerov et al. [6] provided a systematic analysis of the application of HAA in fire protection of farmland, steppe, and forest tracts. The authors argued that the HAA can perform round-the-clock patrolling and response to emergencies along optimal routes in all regions of Russia. Particularly, the HAA is of significance in diagnosing the environment of mountainous areas that are difficult to be monitored due to the limited material and human resources. The Skyship project [7] created by South Korean telecom giant KT Corp has built a 5G-connected airship to help transform search and rescue missions. The Skyship can search for signs of life in disaster areas by using thermal imaging. Cloudline Dynamics [8] dedicates to applying airships in health logistics, precision agriculture, infrastructure monitoring, and wildlife management. In case of a natural disaster, the airships of Cloudline Dynamics can immediately be deployed for logistics, data continuity, and live video feed applications. Besides, the airships “5G Cloud One” [9] and “LanTianHao” [10] have been tested in China for emergency rescue, disaster relief, and traffic monitoring applications in the future.

When an HAA is launched for executing observation tasks in some application cases such as environment monitoring and fire prevention, the HAA is generally required to take numerous pictures from many locations. Therefore, a high-quality observation scheduling algorithm is crucial to improve observation efficiency by determining the observation sequence of target locations. It should be noted that the observation scheduling problem of HAA is distinguished from that of other observation platforms. Specifically, EOS has multiple observation chances during a scheduling horizon since it files along its orbit at a high speed [4]. In contrast, due to the low cruise speed, HAA usually hovers above the ground target for fixed-point observations. Hence, the observation scheduling for HAA is not constrained by orbit issues faced by EOS. Although UAV also can hover above the ground target, the observations are comprised of several dispersed activities restricted by the flight endurance of the UAV. Besides, different from HAA that is not limited by launch and retrieve nodes due to its long flight endurance, the observation scheduling problem of the UAV needs to consider the constraints yielded by launch and retrieve nodes (generally the launch and retrieve nodes are the same) [11].

However, to our best knowledge, a limited number of studies have been carried out to address the observation scheduling problem of HAA [12,13,14]. Besides, the existing studies mainly focused on optimizing the cruise path of HAA and rarely considered the planning of the imaging sensor simultaneously. Since the imaging sensor equipped on the HAA can swim or rotate within a certain angular range and the HAA usually cruises at a low speed, it is possible and more practical for HAA to hover above a specific ground node (i.e., stagnation node) and observe nearby targets through appropriately planned sensor rotations. Thus, this paper investigates the observation scheduling problem considering sensor slewing for HAA. To solve the considered problem, it is natural to cluster all observation tasks into several sets and observe the tasks in each set by slewing the imaging sensor of HAA. Hence, we transform the observation scheduling problem into three sub-problems: task clustering, sensor scheduling, and cruise path planning. In the task clustering problem, all observation tasks need to be clustered into several sets, and each set is associated with a stagnation node. The stagnation node is determined during the clustering process and the HAA hovers above the stagnation node to observe its nearby ground targets. The sensor scheduling problem aims to find a reasonable observation sequence executed by the imaging sensor to accomplish observation. Finally, an appropriate path formed by stagnation nodes is determined in the cruise path planning problem. Note that in this paper we regard a ground target as an observation task.

To facilitate the solving of the problem, the three sub-problems are formulated by classical combinatorial optimization problems. Specifically, since the number of ground targets that can be observed by HAA is affected by the maximum slewing angle of the imaging sensor and fewer cruise distances may lead to less energy consumption, the task clustering problem can be treated as a maximum coverage problem (MCP) [15] that aims to cover all ground targets by using the minimum number of field of views (FOV). The FOV is defined as a circular area on the ground in which targets can be observed by slewing the imaging sensor [16]. As to the sensor scheduling and cruising path planning problems, since the aim of them is to find permutations of nodes, they can be formulated as variants of vehicle routing problems (VRP) [17,18].

We implement heuristic and meta-heuristic algorithms to solve these sub-problems. Specifically, as 2-opt local search and ant colony optimization (ACO) have been proved effective in solving vehicle routing problems [19,20,21], we appropriately combine them with several sophisticated mechanisms into a hierarchical framework to solve the observation scheduling problem of the HAA. In the hierarchical framework, we adopt a heuristic algorithm embedding an improved ant colony optimization (IACO) and a backtracking strategy to solve the task clustering problem as well as find stagnation nodes. A simple but efficient heuristic algorithm combining the 2-opt local search and nearest neighbour search (NNS) is developed to handle the sensor scheduling. Meanwhile, the IACO is also implemented to solve the cruise path planning problem. Particularly, the IACO is developed by hybridizing ACO with local search, as well as modifying the selection criteria of candidate nodes.

Therefore, the contributions of this paper are summarized as follows. (i) We consider the scheduling of the imaging sensor in the observation scheduling of HAA, which is rarely considered in previous studies. (ii) To solve the studied problem, we transform the original problem into three sub-problems and propose an observation scheduling approach based on task clustering (SA-TC). The SA-TC has a hierarchical framework that is composed of three stages corresponding to the three sub-problems. In the first stage, a heuristic algorithm based on a backtracking strategy and IACO is developed to cluster tasks and determine stagnation nodes. In the second stage, a heuristic algorithm that combines the 2-opt local search and NNS is proposed to solve the scheduling problem of the sensor, followed by the third stage in which IACO is implemented to solve the planning problems of the cruise path. Besides, the IACO integrates local search and appropriate candidates selection strategy to improve the performance of ACO. (iii) We conduct extensive experiments to prove the superiority of the proposed approach. Meanwhile, the three algorithms for three sub-problems are further analyzed.

This paper is structured as follows. Section 2 provides a literature review and Section 3 describes the observation scheduling problem. Section 4 introduces the proposed hierarchical scheduling approach based on task clustering. Experimental studies and conclusions are presented in Section 5 and Section 6, respectively.

## 2. Related Work

To better apply the HAA platform, numerous relevant studies have been carried out in recent years. For example, Wang et al. [22] investigated a path-following control method for a robotic airship subject to sensor faults. To detect and isolate the sensor fault, the authors designed a data-driven soft sensor based on an adaptive neuro-fuzzy inference system to detect and isolate the sensor fault. Zhang et al. [23] proposed an energy optimization strategy based on the position potential for airships to improve endurance performance. To improve the solar energy system of solar airships, Zhu et al. [24] studied the altitude planning method by optimizing the yaw angle of airships. Another study conducted by Zhang et al. [25] also focused on the altitude planning problem of solar airships. The authors further analyzed the energy balance using different angle planning strategies. Besides, Wang et al. [26] introduced the recovery trajectory optimization problem of the stratospheric airship for the station-keeping mission and adopted an off-the-shelf nonlinear programming problem solver to address this problem. These above-mentioned studies are utilized to improve the performance of the HAA platform (e.g., reducing energy consumption and easing control). However, few studies have paid attention to the observation scheduling problem, which is one of the most crucial aspects of HAA applications.

Previously, to investigate the scheduling problem of HAA, Zhu et al. [12] proposed a novel agent-based scheduling mechanism for multiple airships oriented to emergencies. The authors integrated a bidirectional announcement mechanism and a collaborative process into the proposed mechanism to facilitate the scheduling. Chuan et al. [13] investigated the cooperative scheduling problem on HAAs for imaging observation tasks. An intelligence algorithm named propagation algorithm combined with the key node search algorithm was proposed by the authors to solve the cruising optimization problem of HAA. Similarly, the same group carried out a further study [14], in which the authors proposed an energy-aware optimization strategy that optimizes task benefits and energy consumption simultaneously. A recent study conducted by Xu et al. [27] developed a decomposition-based multi-objective optimization algorithm using dynamic weight vectors and stable matching schemes to address the deployment problem of airships. Several studies also consider the observation scheduling of airships coordinated with other observation platforms. For example, Deng et al. [28] focused on an Earth observation network consisting of heterogeneous observation resources (e.g., satellites, UAVs, airships, and ground monitoring vehicles), and proposed a two-phase coordinated planning approach to efficiently utilize these observation resources. Wu et al. [29] developed a hierarchical coordinated planning architecture that includes observation resources, sub-planner, coordination, and information management. Meanwhile, two task assignment algorithms were designed by the authors to coordinate and allocate observation tasks to sub-planners. In addition, Wang et al. [30] focused on the coordinated scheduling problem in space information networks. It can be concluded that previous studies mainly focused on optimizing the execution sequence of all tasks to plan a reasonable cruise path for HAA and the sensor slewing is rarely considered. In our study, we integrate the sensor scheduling into the observation scheduling problem of HAA to fill the gap.

As mentioned above, the observation scheduling problem is divided into three sub-problems based on our proposed method. These sub-problems can be formulated by maximum coverage problem (MCP) and variants of vehicle routing problem (VRP), which have been proved to be NP-hard [15,17]. To solve these combinatorial problems, it is commonly believed that exact algorithms cannot find optimal solutions within an acceptable time. Hence, heuristic and meta-heuristic algorithms are widely implemented to obtain approximately optimal solutions. For instance, to address the coverage problem that commonly appears in mobile wireless sensor networks, Liang et al. [31] devised four heuristic algorithms and validated the effectiveness of the proposed algorithms. Chauhan et al. [32] proposed a robust three-stage heuristic to solve the coverage facility location problem with drones considering uncertainties such as battery availability and consumption. A recent study conducted by Li et al. [33] solved a new variant named budgeted maximum coverage problem based on an algorithm that combines reinforcement learning with tabu search. For solving VRP and its variants, efficient algorithms such as tabu search [34,35], variable neighborhood search [36], large neighborhood search [37], genetic algorithms [38], iterated local search algorithms [39,40], and hybrid algorithms [41,42] have been adopted in existing studies. However, the existing heuristic and mate-heuristic algorithms cannot be directly implemented to solve our studied problem. Thus, we carry out some problem-specific modifications as well as heuristic algorithms are required to obtain high-quality solutions.

## 3. Problem Description

**Figure 1:**
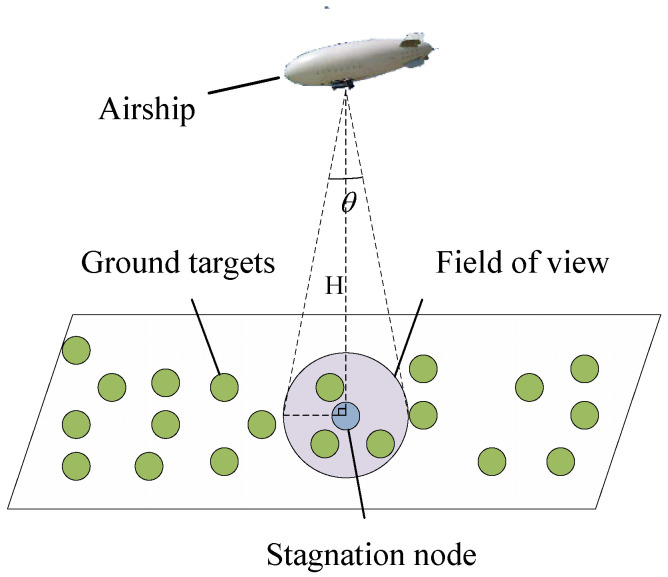
An example of the HAA observation process.

As shown in Figure 1, during the observation period, an airship flies along with its cruise path and begins to observe by hovering above a stagnation node. The ground targets nearby the stagnation node are covered by the airship’s field of view (FOV) and these targets are observed by slewing the imaging sensor equipped on the airship. Further, the cruise path of the airship is formed by a set of stagnation nodes to accomplish all observations. Note that each stagnation node is either at the location of a ground target, or a new location determined by multiple ground targets in a FOV.

To facilitate the problem description and modeling, some reasonable assumptions are given as follows. (i) During the observation, the impacts of sensor resolution and clouds are assumed to be negligible. (ii) The radius of the Earth’s curvature is not considered in this study. (iii) The working height of the airship is assumed to be maintained, thus this paper only considers the horizontal movement of the airship. (iv) Assume that an airship is equipped with an imaging sensor and the sensor can rotate freely but is constrained by its maximum slewing angle. Besides, the notations used in this section are summarized in Table 1.

As mentioned above, the observation process of HAA includes two parts: (i) the airship flies along with the cruise path formed by stagnation nodes, and (ii) the airship hovers above each stagnation node and rotates the imaging sensor to observe the ground targets covered by the FOV. Hence, to accomplish all tasks efficiently, the optimization objective of the observation scheduling problem is the total observation time, which can be written as

(1) $$f=min\sum_{i,j\in S}x_{ij}\alpha_{ij}+\sum_{u\in C}\sum_{k,l\in T}z_{ku}z_{lu}y_{kl}\beta_{kl},$$

where the first part of the objective function represents the total time for the airship flying along the cruise path and the second part represents the total time required by the imaging sensor to accomplish all observation tasks in all clusters. Here each cluster is formed by one or multiple ground targets, which is determined by the FOV of the airship hovering above every stagnation node.

Besides, the main constraints that should be satisfied are as follows.

(2) $$\sum_{i\in S}x_{ij}\leq 1,\;\forall j\in S,$$

(3) $$\sum_{k\in T}y_{kl}\leq 1,\;\forall l\in T,$$

(4) $$\sum_{u\in C}z_{iu}\leq 1,\;\forall i\in T,$$

(5) $$arccos\frac{\delta_{k}^{2}+\delta_{l}^{2}-d_{kl}}{2\delta_{k}\delta_{l}}+M\left(1-y_{kl}\right)\leq \theta ,\forall k,l\in \left\{u:k\neq l,u\in C\right\},$$

(6) $$d_{ij}+M\left(1-z_{iu}\right)\leq H\left(tan\frac{\theta }{2}\right),\forall i\in T,\forall j\in S,\forall u\in C,$$

(7) $$x_{ij}\in 0,1,\forall i,j\in \left\{S:i\neq j\right\},$$

(8) $$y_{kl}\in 0,1,\forall k,l\in \left\{T:k\neq l\right\},$$

(9) $$z_{iu}\in 0,1,\forall i\in T,u\in C.$$

Constraint (2) ensures that each stagnation node should be visited by the airship only once. Similarly, constraint (3) represents that each ground target should be observed only once. Constraint (4) guarantees that each ground target only can be collected by a cluster. Constraint (5) indicates that the slewing angle of the imaging sensor used for observing two successive ground targets in a cluster should not exceed the maximum slewing angle. In constraint (6), the condition for a ground target *i* being observed by the airship hovering above stagnation node *j* is presented. Constraints (7)–(9) define the decision variables.

The above problem descriptions indicate that it is difficult to solve the studied observation scheduling problem directly as the problem includes three decision variables and complicated constraints. Hence, in this paper, we transform the original problem into three sub-problems, including task clustering, sensor scheduling, and cruise path planning. The task clustering problem can be treated as a MCP [15] that aims to cover all ground targets by using the minimum number of field of views (FOV). Since the result of the sensor scheduling is the observation sequence of ground targets and the cruise path of the airship is formed by stagnation nodes, the sensor scheduling and cruise path planning problems can be regarded as two variants of VRP [17,18]. Thus, the original scheduling problem can be decomposed and then solved in a step-by-step manner.

## 4. Observation Scheduling Approach Based on Task Clustering

The proposed scheduling approach has a hierarchical framework composed of two heuristic algorithms and one meta-heuristic algorithm: the heuristic algorithm for task clustering (HATC), the heuristic algorithm for sensor scheduling (HASS), and the improved ant colony optimization (IACO). We call the proposed approach SA-TC. The framework of SA-TC as well as its components are detailed in this section.

### 4.1. Framework of the Proposed Approach

**Figure 2:**
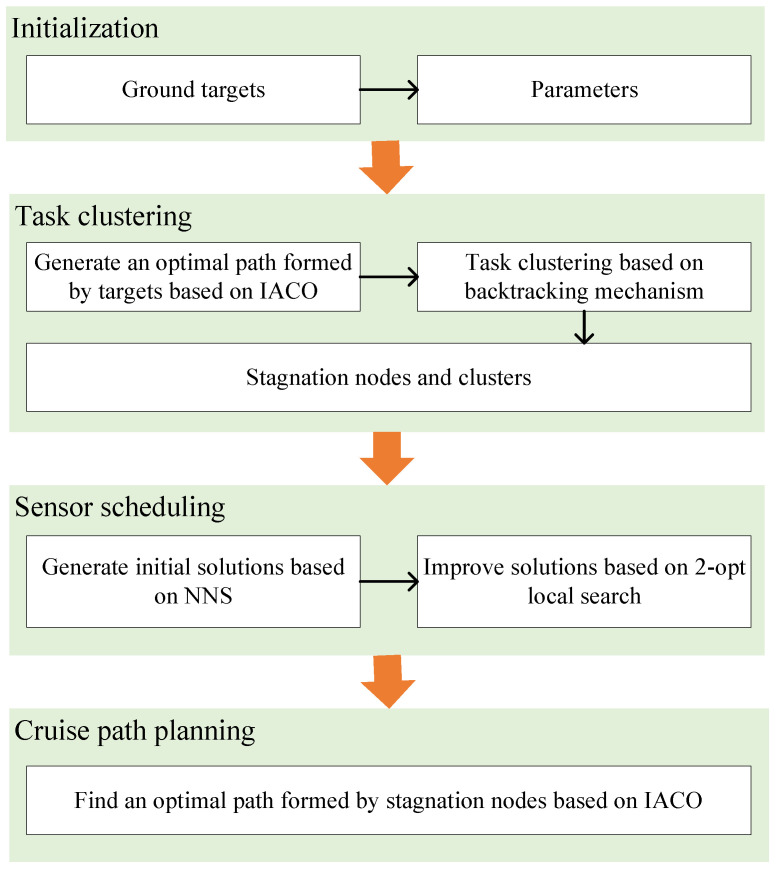
The framework of the proposed observation scheduling approach.

The framework of SA-TC is shown in Figure 2, in which we adopt three algorithms to solve three sub-problems as mentioned above. More specifically, we develop a heuristic algorithm named HATC to divide all observation tasks into several sets (i.e., cluster). First, an optimal path formed by all tasks is generated based on IACO. Then, a backtracking strategy is introduced to cluster tasks along the optimal path. Meanwhile, each cluster is associated with a stagnation node determined by the locations of the tasks in the cluster. By assuming that the airship hovers above the stagnation node to accomplish observations, a heuristic algorithm named HASS is proposed to search for a reasonable task observation sequence in the sensor scheduling stage. The initial solution of the HASS is generated based on the nearest neighbour search (NNS) and improved by 2-opt local search. Finally, all stagnation nodes are used to form an appropriate cruising path for the airship based on IACO.

### 4.2. Heuristic Algorithm for Task Clustering

As mentioned above, the task clustering problem can be regarded as a maximum coverage problem whose objective is to minimize the number of task clusters. Here a cluster is defined by a set of ground targets that can be observed by the airship hovering above a stagnation node. We propose a heuristic algorithm based on the improved ant colony optimization (detailed in Section 4.4) and backtracking strategy for clustering tasks, namely HATC, to determine the clusters and stagnation nodes. The main steps of HATC are illustrated in Algorithm 1.

In HATC, the first step is to form an optimal path consisting of all ground targets by using IACO. Then, the backtracking strategy is performed in lines 2–34 to cluster tasks as well as determine stagnation nodes. More specifically, we denote the first two targets along the optimal path as *a* and *b*, and set a stagnation node *o* at the middle position between *a* and *b* (lines 3–4). If both *a* and *b* can be observed by the airship hovering above *o* according to constraint (6), both *a* and *b* are clustered into the set ctask; otherwise, only put *a* into ctask and move *o* to the position of *a* (lines 5–9). Afterward, two operations are conducted to add feasible targets into ctask. In the first operation (lines 10–22), the set of the remained targets that are not clustered is defined as UT and the closest target *c* from UT to the stagnation node is selected in each iteration. Assume a stagnation node $o^{\prime }$ at the middle position between *c* and *o*, if target *c* and other targets in ctask can be simultaneously observed by the airship hovering above $o^{\prime }$ according to constraint (6), target *c* would be added into the set ctask, and the stagnation node as well as the remained target set UT would be updated. Similarly, the second operation (lines 23–33) also tries to add the remained feasible targets into the set ctask. However, different from the first operation in which the candidate tasks are selected greedily, the second operation stochastically selects candidate targets to help the algorithm escape from the local optimum.
**Algorithm 1:** HATC.**Input**: All ground targets**Output**: clusters and stagnation nodes
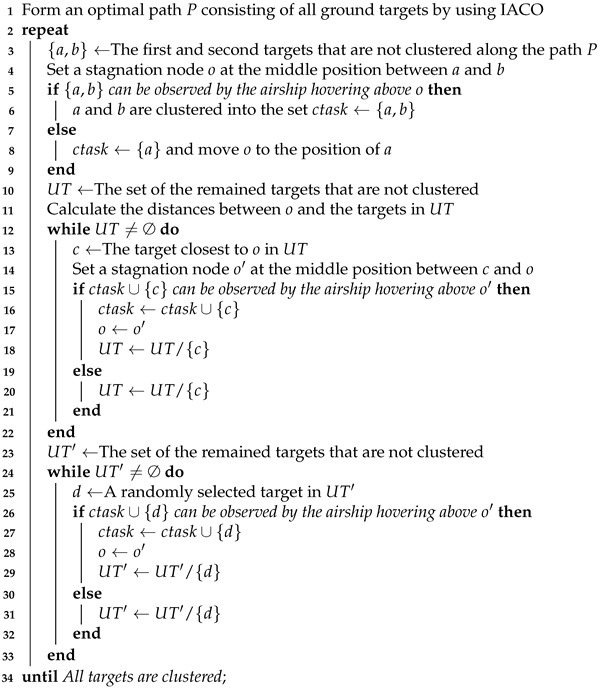


**Figure 3:**
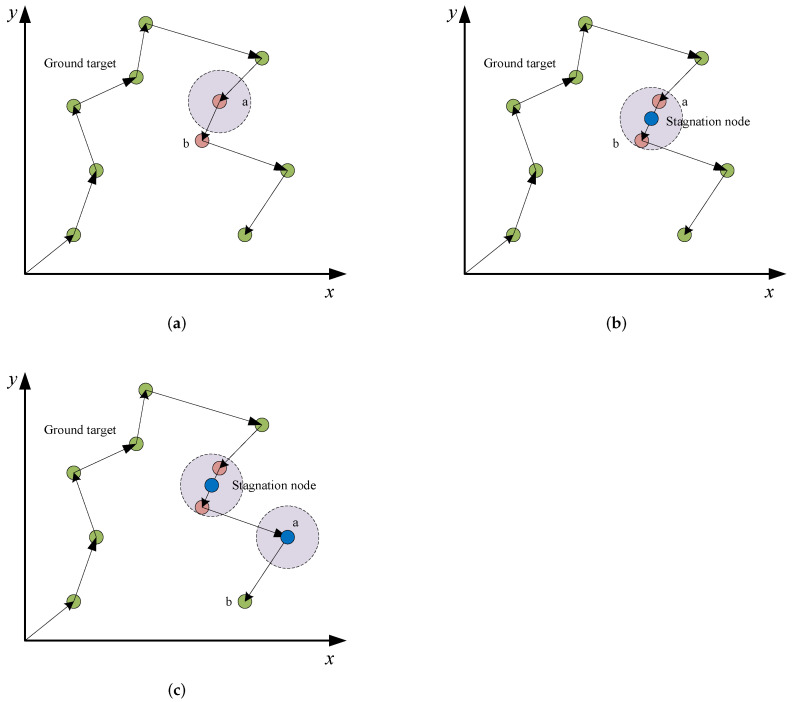
An illustration of the task clustering process. (**a**) A path formed by all ground targets, (**b**) A path with a stagnation node, (**c**) A path with two stagnation nodes.

To better understand the backtracking strategy, as well as the proposed HATC, we present an example in Figure 3. In Figure 3a, there is an optimal path formed by all ground targets (i.e., observation tasks), and the ground target *b* is successive to the ground target *a* along the optimal path. As Figure 3b shows, if a stagnation node is located at the middle position between *a* and *b*, and the FOV provided by the stagnation node can cover *a* and *b* simultaneously, *a* and *b* would be clustered into the same set. Afterward, for the case in Figure 3c, the FOV cannot cover the ground target *b* if the stagnation node is located at the middle position between *a* and *b*. Then, the position of the stagnation node would be moved back to the position of ground target *a*. The output of HATC is a set of clusters covering all ground targets and a set of stagnation nodes. Meanwhile, each cluster is associated with a feasible stagnation node.

### 4.3. Heuristic Algorithm for Sensor Scheduling

To reduce the time for slewing the imaging sensor, it is necessary to optimize the observation sequence of ground targets. The sensor scheduling problem in each cluster can be regarded as a traveling salesman problem (TSP), in which the observation sequence is represented by a path consisting of all ground targets in a cluster and the stagnation node is regarded as the depot node. Meanwhile, due to the angle constraint of the imaging sensor as mentioned in constraint (5), two ground targets may not be observed successively, yielding difficulties for solving this problem. Hence, since the number of ground targets in each cluster is limited, we propose a simple but efficient heuristic algorithm for sensor scheduling (HASS) to find a reasonable observation sequence of ground targets in each cluster. The main steps of HASS are displayed in Algorithm 2.

**Figure 4:**
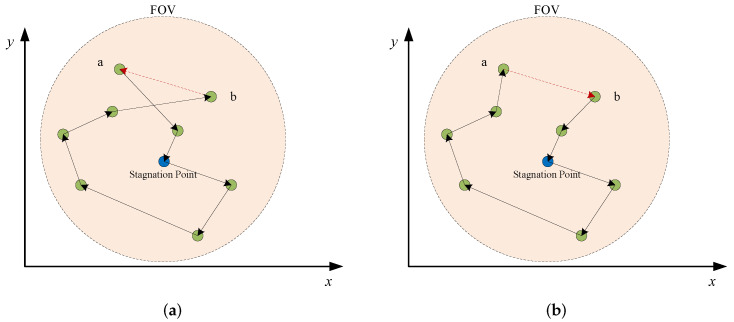
An illustration of the 2-opt operation. (**a**) A path before 2-opt operation, (**b**) A path after 2-opt operation.

As illustrated in HASS, the well-known nearest neighbour search (NNS) is adopted to create an initial observation sequence (i.e., a path composed of ground targets) for each cluster (line 2). From the stagnation node, the NNS chooses the next target that is the closet target from unvisited targets and constrained by the maximum slewing angle of the sensor, finally stops when all targets are visited. Then, the 2-opt local search is adopted to improve the initial solution in lines 4–11 as 2-opt based local search has been proved effective in solving various routing problems [43,44]. In each iteration of the 2-opt local search, two nodes would be randomly selected from the path and the sub-path between these two nodes would be reversed (i.e., 2-opt operation). An example of the 2-opt operation is illustrated in Figure 4. Figure 4a shows an observation sequence of ground targets in a cluster. Then, based on the 2-opt operation, targets *a* and *b* are selected and the sub-path between *a* and *b* is reversed to construct a better path, as Figure 4b shows. It should be noted that we implement a repair strategy to address possible infeasibilities caused by the slewing angle constraint. Specifically, if a solution generated by a 2-opt operation is infeasible in line 5, this step would output the solution unchanged. Finally, the algorithm stops when the path in every cluster is optimized by the local search.
**Algorithm 2:** HASS.**Input**: clusters *C* and stagnation nodes *S***Output**: observation sequences of the sensor
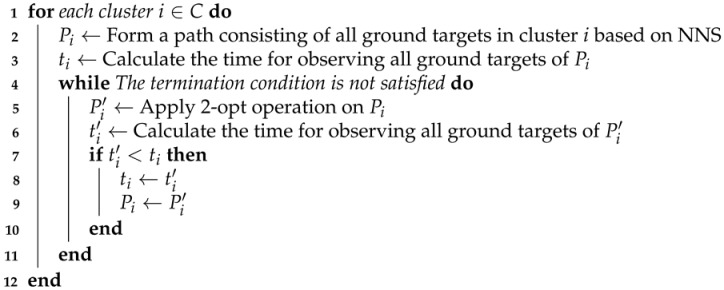


### 4.4. Improved ant Colony Optimization

Ant colony optimization (ACO) is a bio-inspired swarm intelligence algorithm that has been applied in many fields due to its powerful search ability [19,45,46]. At each iteration, when an ant travels through an edge between two nodes, it will deposit some pheromone trail on this edge. Besides, the quantity of the pheromone depends on the quality of the solution. Thus, each ant *k* selects the next node *j* from the current node *i* based on a specific probability $p_{ij}^{k}$ determined by the pheromone concentration, which can be written as

(10) $$p_{ij}^{k}=\left\{\begin{array}{cc} \frac{\tau_{ij}^{\alpha }\cdot \eta_{ij}^{\beta }}{\sum_{l\in J_{k}\left(i\right)}\tau_{il}^{\alpha }\cdot \eta_{il}^{\beta }}, & \mathrm{if}\;j\in J_{k}\left(i\right),\\ 0, & \mathrm{otherwise}, \end{array}\right.$$

where $J_{k}\left(i\right)$ is the set of feasible nodes unvisited by the ant *k*, α and β are weight coefficients that show the relative importance of the pheromone trail $\tau_{ij}$ and the heuristic information $\eta_{ij}$. Particularly, the heuristic information $\eta_{ij}$ is generally defined by

(11) $$\eta_{ij}=\frac{1}{d_{ij}},$$

where $d_{ij}$ is the distance between nodes *i* and *j*. Besides, the pheromone trail $\tau_{ij}$ would be updated after every ant constructs a complete path. The update methods are described as follows:

(12) $$\tau_{ij}=\left(1-\rho \right)\cdot \tau_{ij}+\sum_{k=1}^{m}\Delta \tau_{ij}^{k},$$

(13) $$\Delta \tau_{ij}^{k}=\frac{Q}{L_{k}},$$

where ρ is the pheromone trail evaporating rate, and $\Delta \tau_{ij}^{k}$ is the amount of pheromone trail accumulated on the path $\left(i,j\right)$ by the ant *k*, *Q* is a constant parameter, and $L_{k}$ is the distance of the path traveled by the ant *k*.

In this paper, we propose an improved ant colony optimization (IACO) to solve the routing problems yielded in the task clustering problem and cruise path planning problem. We improve the ACO from two aspects. Specifically, we appropriately integrate a 2-opt-based local search into ACO to improve its exploration capability. On the other hand, different from the conventional ACO in which all unvisited nodes are considered as candidate nodes when the ant selects the next node, the ant of IACO only selects from the *n* closest nodes, thereby improving the convergence efficiency. The main steps of IACO are illustrated in Algorithm 3.
**Algorithm 3:** IACO.**Input**: *N* nodes, the maximum number of iterations *G*, the number of ants *m*, α, β, ρ, and *Q***Output**: Best path $P^{best}$
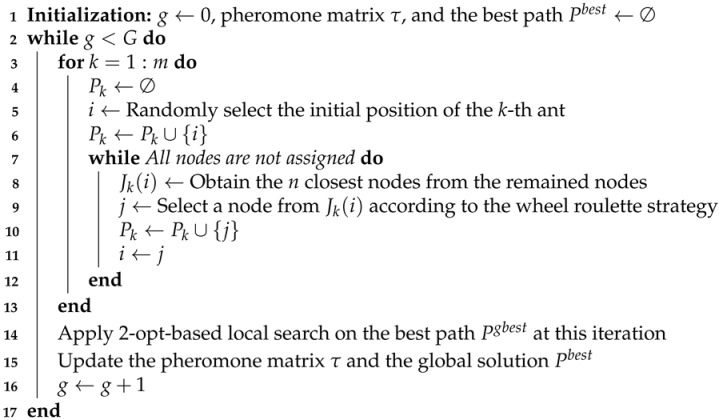


As Algorithm 3 shows, the inputs of IACO are the nodes, the maximum number of iterations *G*, the number of ants *m*, and other parameters mentioned in Equations (10)–(13). At each iteration, each ant starts from a randomly selected node and moves to the next node selected from the remained nodes until all nodes are included in the path (lines 5–12). Particularly, in the proposed IACO, the ant moves from the current node *i* to the next node *j* selected from the *n* nearest nodes based on the wheel roulette strategy (line 8). Note that the value of *n* would change as the number of unvisited nodes decreases. Assume n=5 at the beginning, if the number of the unvisited nodes is more than 5, let n=5; otherwise, *n* equals the number of the unvisited nodes by the ant. The probability of each candidate movement is determined based on Equation (10). After every ant accomplishes a tour, the 2-opt-based local search introduced in Section 4.3 would be applied to further improve the quality of the solution obtained at this iteration. Then, the pheromone information is updated according to Equations (12) and (13) and the best path is recorded as $P^{best}$ (line 15). Finally, the algorithm stops when the maximum number of iterations is met.

## 5. Simulation Experiment and Results

This section represents the simulation and comparative analysis of the proposed approach and its components. As far as we know, there are no accepted benchmarks yet for the studied problem, thus we generate targets randomly distributed in a 500 km × 500 km area to simulate the experiment scenarios. Besides, all algorithms are coded in MATLAB and run on a PC with Core i5-8400 2.80 GHz CPU, 8G memory, and Windows 10 operating system.

### 5.1. Simulation Setup

In experimental studies, we use 6 cases with different target numbers to simulate small-, medium-, and large-scale scenarios, respectively. The HAA is assumed to work at a height of 23 km with an average speed of 500 km/h. The maximum side slewing angle of the imaging sensor is set to $25^{\circ }$ and the slewing speed is set to $5^{\circ }$/s. According to previous experiments, the parameters of the IACO algorithm are set as follows: the number of ants m=50, the weight coefficients α=5 and β=1, the evaporation rate ρ=0.9, the parameter Q=100, and the maximum number of iterations G=100. Besides, the maximum number of iterations of 2-opt-based local search is set to $n^{2}$, where *n* is the number of targets.

Furthermore, in all comparison experiments, we use a metric called GAP in addition to the optimization objective to represent the difference between the results (i.e., $f_{a}$ and $f_{b}$) of two algorithms. The GAP is calculated by

(14) $$GAP\left(a,b\right)=\frac{\left(f_{a}-f_{b}\right)}{max(f_{a},f_{b})}\times 100\%$$

### 5.2. Comparative Study of the Proposed Approach

**Figure 5:**
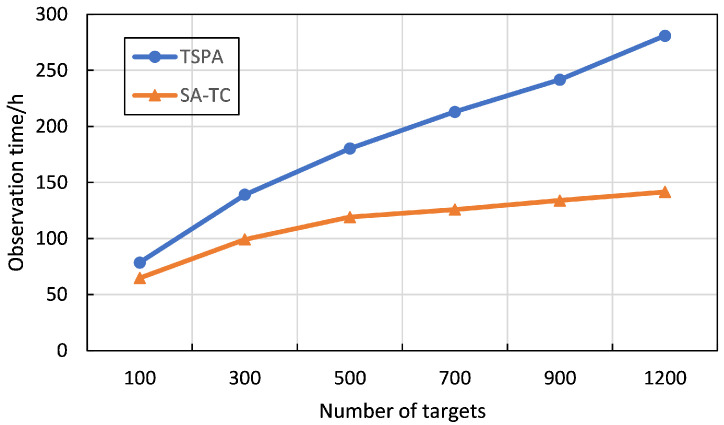
Comparison results of TSPA and SA-TC with different numbers of targets.

To verify the superiority of the proposed SA-TC, we compare it with the classical airship observation scheduling approach [47], namely TSPA, in 6 cases with the number of targets varying from 100–1200. In TSPA, the observation process of HAA is assumed to be a TSP, in which the imaging sensor scheduling and stagnation nodes are not considered. The airship observes all targets based on its cruising ability. During the simulations, each experiment is repeated 10 times and the average observation time is used for comparisons. The experimental results are displayed in Table 2 and Figure 5. In Table 2, the colonums represent the number of targets, the observation time results obtained by TSPA and SA-TC respectively, the number of clusters obtained by SA-TC, and the values of GAP(TSPA,SA-TC) calculated by Equation (14).

The results demonstrate that the proposed SA-TC is superior to TSPA in all cases. Specifically, according to the values of GAP(TSPA,SA-TC), the proposed SA-TC can reduce the total observation time by 17.51% to 49.61% compared with TSPA. Besides, as the number of targets increases, the superiority of SA-TC becomes more significant. For instance, when the number of targets is 500, the observation time required by SA-TC is 66.67% of the time required by TSPA. When the number of targets is 1200, the observation time required by SA-TC is 50% of the time required by TSPA. Therefore, the proposed SA-TC is more efficient when dealing with large-scale observation scheduling problems. The superiority of SA-TC can be explained by the task clustering strategy. Based on the task clustering strategy, the airship only needs to hover above specific stagnation nodes to observe nearby targets, thereby reducing the cruise time of the airship.

**Figure 6:**
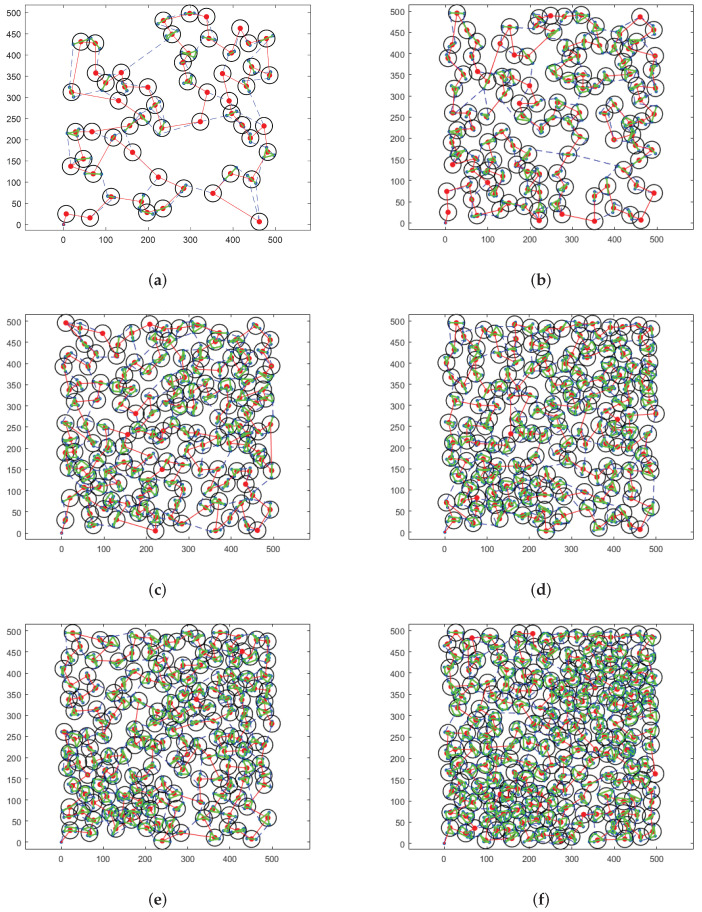
Scheduling results of TSPA and SA-TC. (**a**) 100 targets, (**b**) 300 targets, (**c**) 500 targets, (**d**) 700 targets, (**e**) 900 targets, (**f**) 1200 targets.

To visually compare the scheduling results, we present the experimental results in Figure 6. In Figure 6, the red node indicates the stagnation node, the black circle denotes the FOV of the airship hovering above the stagnation node, the green line represents the observation sequence of the imaging sensor, and the red line is the cruise path of the airship obtained by SA-TC. Besides, the cruise path of the airship obtained by TSPA is represented by the blue dotted line. It can be found that the proposed SA-TC can well address the cases with different scales of targets.

### 5.3. Performance Analysis of HATC

This section analyzes the performance of the proposed HATC by conducting experiments in cases with the number of targets varying from 30–400. Each experiment is repeated 10 times and the average results are displayed in Table 3. The results indicate that the proposed HATC can handle different scales of targets to obtain clusters and stagnation nodes. Besides, although the CPU time increases with the increase of the number of targets, the time consumption is still acceptable.

**Figure 7:**
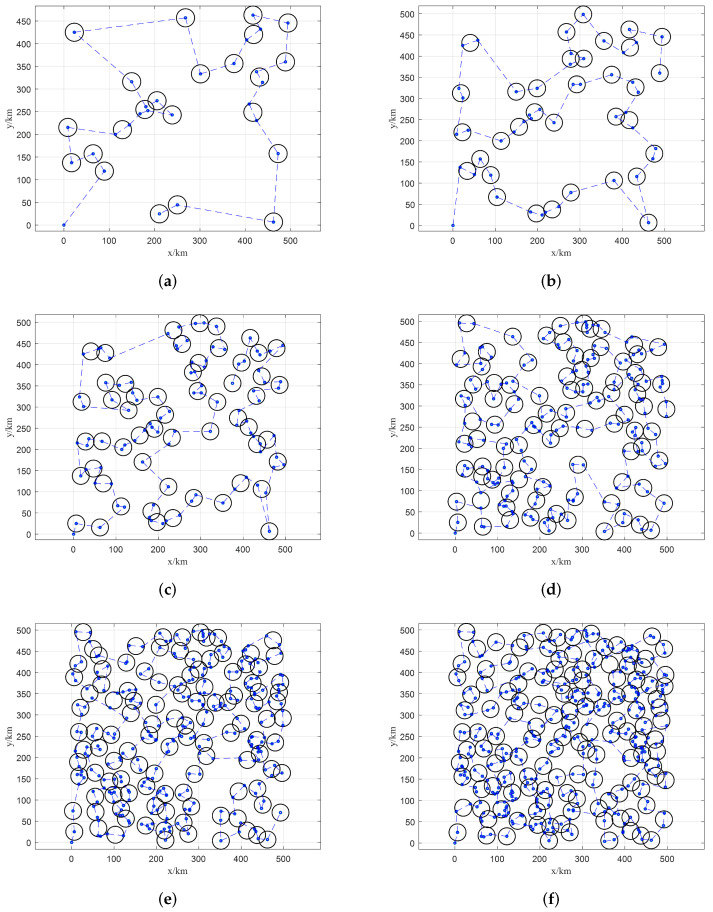
Scheduling results of HATC with different numbers of targets. (**a**) 30 targets, (**b**) 50 targets, (**c**) 100 targets, (**d**) 200 targets, (**e**) 300 targets, (**f**) 400 targets.

The visual results of HATC are shown in Figure 7. The blue dotted line is the path obtained by IACO, the blue nodes are stagnation nodes obtained by the backtracking strategy, and the black circle is the range of the cluster. An interesting result is that the increment of the number of clusters generated by the algorithm is decreasing as the number of targets increases. It can be explained that with the increase of the target number, the density of targets would be increased, which would also yield difficulty for obtaining a reasonable solution. Nevertheless, the large-scale case is well-addressed by the proposed HATC.

### 5.4. Performance Analysis of HASS with Different Numbers of Targets

**Figure 8:**
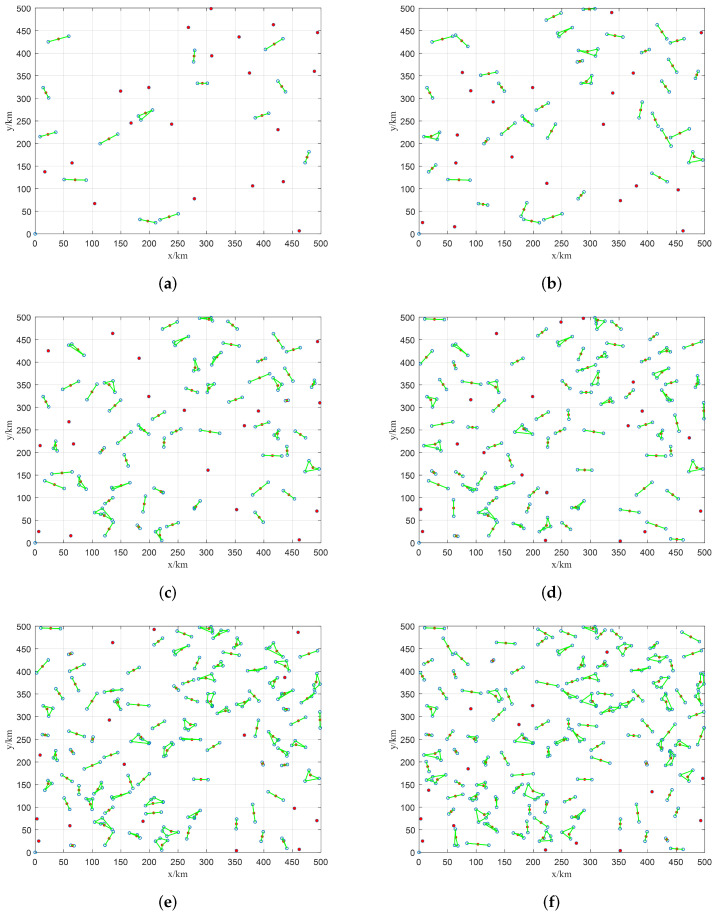
Scheduling results of HASS with different numbers of targets. (**a**) 50 targets, (**b**) 100 targets, (**c**) 150 targets, (**d**) 200 targets, (**e**) 250 targets, (**f**) 300 targets.

In this section, we set 6 experimental cases with the number of targets varying from 50–300 to investigate the performance of the proposed HASS. The simulation results of HASS are displayed in Figure 8. The green line, red node, and blue circle represent the observation sequence of the imaging sensor, stagnation node, and target, respectively. It should be noted that the slewing path of the imaging sensor is a closed-loop, and the start and termination nodes of the path are the same stagnation node, thus the slewing direction of the imaging sensor is not provided in Figure 8. As it can be seen, almost every sensor scheduling problem in each cluster is well-scheduled. Besides, every sensor scheduling problem in each cluster contains a very small number of nodes (generally less than 5 as shown in Figure 8), thus it can be solved quickly by using 2-opt-based local search. Also, since it is very easy to obtain an optimized solution on such a small-scale problem, no further performance evaluation of HASS is provided in this section. This result also demonstrates the application of the 2-opt-based local search is reasonable for solving the sensor scheduling problem. Furthermore, as discussed before, the density of nodes increases with the increase of target number, so the problem scale of each sensor scheduling problem in every cluster is also increased. Nevertheless, it can be found from Figure 8f that the scale of the sensor scheduling problem is still moderate to be solved by HASS.

### 5.5. Performance Analysis of IACO

**Figure 9:**
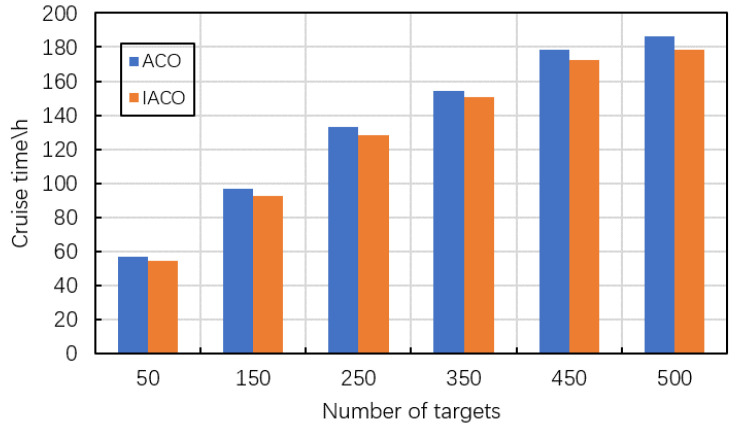
Cruise time of the airship in 6 cases with different numbers of targets obtained by HASS.

This section compares the IACO with conventional ACO to verify the efficiency of the proposed IACO. The experiments are conducted in 6 cases whose scales are 50, 150, 250, 350, 450, and 500, respectively. Each experiment is repeated 10 times and the average results are used for comparisons. The results are shown in Table 4 and Figure 9. In Table 4, the columns indicate the number of targets, the cruise time obtained by ACO and IACO respectively, the CPU time consumed by ACO and IACO respectively, and the values of GAP(ACO,IACO). It can be found that the proposed IACO can obtain better results compared with ACO. Specifically, according to the values of GAP(ACO,IACO), the IACO can reduce the cruise time of the airship by 2.36–4.61% compared with the conventional ACO. This result is attributed to the local search adopted in IACO. By appropriately using the local search, IACO can obtain higher-quality solutions. Meanwhile, the IACO used less computational time. The IACO can reduce the computational time for dealing with the same case by 24.67% at most. It can be explained by that the candidate selection strategy implemented in IACO reduces the search time.

Besides, the impacts of the target number on the IACO are worth the attention. As it can be found in Table 4, with the increase of node number, the computational time of IACO is significantly increased, which is very common for swarm-based meta-heuristic algorithms. However, the time consumption is acceptable for observation scheduling of HAA under the consideration of its long-period observation requirement.

## 6. Conclusions

In this paper, we consider the scheduling of the imaging sensor in the observation scheduling problem of HAA. To solve the studied problem, we propose a hierarchical observation scheduling approach based on task clustering. The proposed approach transforms the original observation scheduling problem into three sub-problems: task clustering, sensor scheduling, and cruise path planning, and solves these sub-problems in a step-by-step manner. In the task clustering problem, a heuristic algorithm named HATC is proposed to cluster ground targets and find a set of stagnation nodes. Each stagnation node is associated with a cluster, and the airship hovers above the stagnation node to observe nearby ground targets. To observe the targets nearby the stagnation nodes, a heuristic algorithm named HASS is developed to schedule the observation sequence of the imaging sensor in the sensor scheduling problem. Finally, a meta-heuristic algorithm named IACO is designed to obtain a cruise path of the airship that connects all stagnation nodes in the cruise path planning problem.

Besides, some sophisticated mechanisms are adopted to design the algorithms. Specifically, the HATC integrates the IACO to generate an initial solution, and the backtracking strategy to determine clusters and stagnation nodes. Since the observation sequence scheduling in each cluster is small-scale, the 2-opt local search combined with nearest neighbour search is used to develop the HASS. The IACO proposed in this paper appropriately combines local search and modifies the selection strategy of candidate nodes to improve the performance of the conventional ACO.

The simulation results of this paper show that the proposed approach can reduce the total observation time by at most 49.61% compared with the traditional airship observation method. Particularly, the proposed approach is more efficient when dealing with large-scale problems. Additionally, the performance of three algorithms in the cases with different scales of targets are analyzed. The results show that all three algorithms can well address small-, medium-, and large-scale problems.

The proposed algorithm is expected to be used in the HAA-based platform for observation tasks in emergencies. For example, numerous observation information and pictures about the disaster area are badly needed when an earthquake occurs. The HAA can be launched to observe a set of geological accident points that cannot be reached by humans, while the proposed algorithm would provide the HAA with an efficient observation scheme. However, the proposed algorithm is not verified by using the benchmark derived from the practical dataset and ignores some constraints that may appear in practical applications (e.g., uncertainties and timeless), which would be addressed in future works.

## Figures and Tables

**Table 1:** Notations.

Parameters	Description
*T*	Set of ground targets
*S*	Set of stagnation nodes
*C*	Set of clusters
$\alpha_{ij}$	The time for the airship cruising from stagnation node i∈S to stagnation node j∈S
$\beta_{kl}$	The time for the imaging sensor rotating from the position observing target k∈T to the position observing target l∈T
$d_{ij}$	Distance between node *i* and node *j* on the ground
$\delta_{i}$	Distance between the airship and target i∈T
*H*	The working height of the airship
θ	The maximum slewing angle of the imaging sensor
*M*	A sufficiently large number
Decision variables	Description
$x_{ij}$	$x_{ij}=1$ if the airship cruises from stagnation node i∈S to stagnation node j∈S; otherwise, $x_{ij}=0$
$y_{kl}$	$y_{kl}=1$ if the sensor rotates from the position observing target k∈T to the position observing target l∈T; otherwise, $y_{kl}=0$
$z_{iu}$	$z_{iu}=1$ if ground target i∈T belongs to cluster u∈C; otherwise, $z_{iu}=0$

**Table 2:** Comparison results of airship observation scheduling approaches in 6 cases.

Case	Num. of Targets	TSPA	SA-TC		GAP (%)
		Obs. Time (h)	Obs. Time (h)	Num. of Clusters	
1	100	78.53	64.78	56	17.51
2	300	139.02	99.18	116	28.66
3	500	180.34	119.11	149	33.95
4	700	212.98	125.82	166	40.92
5	900	241.67	133.92	182	44.59
6	1200	280.85	141.52	200	49.61

**Table 3:** Task clustering results with different numbers of targets.

Case	Num. of Targets	Num. of Clusters	CPU Time (s)
1	30	23	0.02
2	50	34	0.03
3	100	55	0.06
4	200	88	0.10
5	300	114	0.13
6	400	135	0.19

**Table 4:** Comparison results between ACO and IACO in 6 cases.

Case	Num. of Targets	ACO		IACO		GAP (%)
		Cruise Time (h)	CPU Time (s)	Cruise Time (h)	CPU Time (s)	
1	50	57.09	4.50	54.46	3.39	4.61
2	150	96.71	14.39	92.42	15.21	4.44
3	250	132.93	37.67	128.15	35.36	3.59
4	350	154.07	72.57	150.44	64.80	2.36
5	450	178.43	120.90	172.25	103.16	3.46
6	500	186.04	153.46	178.40	120.31	4.11

## Data Availability

Not applicable.
